# Cases of typhoid fever in Copenhagen region: a retrospective study of presentation and relapse

**DOI:** 10.1186/1756-0500-6-315

**Published:** 2013-08-11

**Authors:** Freja Cecille Barrett, Jenny Dahl Knudsen, Isik Somuncu Johansen

**Affiliations:** 1 Department of Infectious Diseases, Copenhagen University Hospital, Hvidovre Hospital, Hvidovre, Denmark; 2 Department of Clinical Microbiology, Copenhagen University Hospital, Hvidovre Hospital, Hvidovre, Denmark; 3 Department of Infectious Diseases, Odense University Hospital, Odense, Denmark

**Keywords:** Typhoid fever, Enteric fever, Travellers, Ciprofloxacin resistance, Relapse

## Abstract

**Background:**

Typhoid fever is a systemic illness which in high-income countries mainly affects travellers. The incidence is particularly high on the Indian subcontinent. Travellers who visit friends and relatives (VFR) have been shown to have a different risk profile than others. We wished to identify main characteristics for travellers infected with *S.* Typhi considering both clinical and laboratory findings in order to provide for faster and better diagnostics in the future. The outcome of treatment, especially concerning relapse, was evaluated as well.

**Methods:**

Retrospectively collected data from 19 adult cases of typhoid fever over a 5-year period at the Department of Infectious Diseases, Copenhagen University Hospital, Hvidovre Denmark.

**Results:**

The patients were young adults, presenting with symptoms within a month after travelling. 84% were returned from travelling in the Indian subcontinent. 17 out of 19 patients were VFR-travellers. The main symptoms were fever (100%), gastrointestinal symptoms (84%), headache (58%) and dry cough (26%). Laboratory findings showed elevated C-reactive protein (CRP) and lactate dehydrogenase (LDH) in all cases and elevated alanine transaminase (ALAT) in 47% of cases. In primary cases 4 isolates were fully susceptible to ciprofloxacin, the remaining were intermediate susceptible. Relapse occurred in 37% of the cases and only in cases where the patient was infected by a strain with intermediate susceptibility.

**Conclusions:**

Better pre-travel counselling should be given to VFR-travellers. The main symptoms and laboratory findings confirm previous findings. The relapse rate was unexpected high and could be correlated to ciprofloxacin-resistance.

## Background

Typhoid fever is a systemic infectious disease caused by *Salmonella enterica* ssp. *enterica* serovar Typhi. (*S.* Typhi). World Health Organization estimates that *S.* Typhi worldwide causes 22 million (16 mill. - 33 mill.) illnesses and 200.000 deaths annually [1]. In endemic areas typhoid fever is an illness primarily affecting children and young adults whereas in high-income countries it is mainly a disease of returning travellers [2-4]. In Denmark, most reported cases today can be related to travelling. In 2008–2010 17–21 cases were reported each year [5]. In the period 1986–1990 the average was 21 [2].

The disease only affects humans and is transmitted through contaminated food or water. Therefore, infection is closely related to poor sanitary conditions. Endemic areas include South-East Asia, South America, Africa and certain areas in the former USSR [1]. The incidence is particular high in the Indian subcontinent and has been shown to be 10–29 times higher in India and Pakistan compared to China and Vietnam [6].

The clinical findings are usually uncharacteristic, but traditional findings include fever, relative bradycardia, splenomegaly and rose spots [4]. If left untreated the mortality is between 10-40% compared to 1-4% with treatment [1]. In Denmark mortality is less than 1% [7]. The diagnosis is usually confirmed by cultivating the bacteria from blood or faeces. Blood culture is considered golden standard even though the sensitivity is as low as 80% [8].

When observing travellers it is important to differentiate between travelling tourists and emigrants visiting friends and relatives (VFR) in the home country. Previous studies have shown a different risk profile in this group [2,9-12]. One study found more patients with *S.* Typhi bacteraemia among holiday-travellers than VFR [13], but most studies agree that VFR-travellers carry a greater risk than tourists [9,10]. In one Danish study observing the period 1986–90 more than 50% of the affected individuals belonged to the VFR group [2].

In order to provide better diagnostics and treatment in the future, we studied the clinical presentation, the travel history, the laboratory findings, and the response to treatment for patients with *S.* Typhi bacteraemia, and especially relapse in relation to resistance.

## Methods

We included retrospectively adult cases of typhoid fever from February 2006 till February 2010 treated at the Department of Infectious Diseases, Copenhagen University Hospital, Hvidovre, Hospital, Denmark. The inclusion criterion was the presence of at least one positive blood culture with *S.* Typhi, which was identified through the database in the Department of Clinical Microbiology, Hvidovre Hospital.

Clinical and laboratory findings were retrieved from patient’s records and the various hospital databases. Permission was granted from the Danish Data Protection Agency. Ethical approval was not required for this study according to Danish Law.

All patients were examined with blood-culture drawn shortly after admittance, as a minimum of four flasks, (two aerobic and two anaerobic flasks, BactAlert®, BioMerieux, France), and often additional blood-cultures were performed depending on the clinical presentation of the patients. Stool samples were examined for enteric pathogen bacteria in most patients.

Fever was defined as a temperature >37.5°C.

Relapse was defined as isolation of *S*. Typhi in new blood cultures drawn after the patient had completed the antimicrobial treatment.

To handle the data OpenOffice Calculator version 3.1.1 was used. Numerical data are presented as the mean and range.

## Results

### Demographics and travel history

19 patients were diagnosed with typhoid fever at the Department of Infectious Diseases, Hvidovre University Hospital during the study period. All were young adults with a median age of 26 years (range 15–40) (Table 1).

**Table 1:** Basic characterization of patients and acquisition of infection

	**Number of patients**
Total number	19 (100%)
Women	11 (58%)
Age, median	26 (range 15–40)
VFR- traveller	17 (89%)
Days spent abroad, median	30 (range 14–150)
Days since return to Denmark, median	16 range (0–26)
Days since onset of symptoms, median	6.5 (range 0–26)
Vaccinated against typhoid fever	0 (0%)
Acquisition in Pakistan	14 (74%)
Acquisition in Asia outside Pakistan	3 (16%)
Acquisition in Middle East	1 (5%)
Acquisition in Sub-Saharan Africa	1 (5%)

The patients had been travelling with a median of 30 days (range 14–150 days). All patients developed symptoms within a month (median 16 days, range 0–26 days) after returning to Denmark and were referred to the Department of Infectious Diseases, Hvidovre Hospital.

The time between onsets of symptoms until patients sought medical attention were median 6.5 days (range 2–26 days). None of the patients received vaccination for typhoid fever prior to travelling abroad (Table 1).

All were infected during travelling outside Denmark, the majority in the Indian subcontinent (84%), primarily Pakistan (Table 1). In 17 of the 19 cases, the patients were emigrant VFR-travellers. All of these were infected in the Indian subcontinent.

### Symptoms and laboratory findings

The main complaint was fever. All of the patients had at some point experienced fever, although only 13 (68%) presented fever at admittance. 16 (84%) patients had at least one gastrointestinal symptom (diarrhoea, nausea, abdominal pain and/or constipation), of these 13 had diarrhoea (68%). Eleven patients had headache (58%), and 5 (26%) experienced dry cough (Table 2). There were no reported cases of rose spots or relative bradycardia.

**Table 2:** Clinical characteristics

**Symptoms**	**Number (%)**
Fever	19 (100)
Diarrhoea	13 (68)
Headache	11 (58)
Vomiting	9 (47)
Nausea	8 (42)
Abdominal pain	6 (32)
Dry cough	5 (26)
Joint or muscle pain	4 (21)
Shivering	4 (21)
Constipation	3 (16)

As given in Table 3 the values of CRP and LDH were elevated at admittance in all patients examined. ALAT was elevated in 8 out of 17 cases (47%). The platelet counts were low within the normal range in 13 cases and below in 6. Haemoglobin values were below reference value in 3 cases, the remaining were normal. The total white blood cells counts were normal in 16 patients, but one had low and two had elevated values. Neutrophil counts were normal in 15 cases, low in one and elevated in three cases. Serum sodium was slightly decreased in 12 cases as were Serum potassium in two cases.

**Table 3:** Laboratory results of adults patients with typhoid fever

**Laboratory examinations of blood (normal ranges)**	**Findings as median (range)**
Haemoglobin (7.0–11.0 mmol/L)	7.9 (6.1–10.0)
MCHC^1^ (19.0–22.0 mmol/L)	21.5 (20.0–22.6)
Serum Sodiium (136–146 mmol/L)	135 (129–140)
Serum Potassium (3.5–5.0 mmol/L)	3.8 (3.4–4.8)
Total white blood cell count (3.0–9.0 10E9/L)	6.1 (2.7–14.2)
Neutrophil count (1.8–7.4 10E9/L)	4.1 (1.6–11.8)
Platelet count (150–400 10E9/L)	176 (112–292)
ALAT^2^ (Male 10–70 U/L; Female 10–45 U/L)	50 (20–121)
LDH^3^ (105–205 U/L)	367 (223–976)
Creatinine (Male 60–130 μmol/L; Female 40–110 μmol/L)	75 (61–107)
CRP^4^ (<10 mg/L)	73.5 (20–199)

^1^*MCHC* mean cell haemoglobin concentration;

^2^*ALAT* alanine transaminase;

^3^*LDH* lactate dehydrogenase;

^4^*CRP* C-reactive protein.

All patients were examined and found negative for malaria infections. Faeces examination at admittance was performed in 13 of the primary cases, and in 3 of the 6 cases of relapses. *S.* Typhi was cultured from faeces in 5 primary cases, and in one secondary case.

All strains were examined for susceptibility to ampicillin, ceftriaxone, sulfonamid-trimethoprim, piv-mecillinam, and ciprofloxacin. None of the isolates were tested for azithromycin and none isolates were multi drug resistant, as defined as resistance to ampicillin, chloramphenicol and sulfonomid-trimethoprim. Ciprofloxacin susceptibility testing was performed in all primary and secondary isolates, using E-tests, and MIC ≤ 0.06 μg/mL were fully susceptible, MIC 0.125 – 1 μg/mL were intermediate susceptible, and > 1 μg/mL were regarded resistant. In primary cases, 4 isolates were fully susceptible, and the rest were intermediate susceptible to ciprofloxacin. In all cases of relapse a similar isolate were found regarding the antibiogram, except for one case, where both the primary ciprofloxacin intermediate resistant isolate was found together with a fully susceptible isolate.

### Treatment and outcome

All patients were hospitalized for a median of six days (1–14 days). Empiric antimicrobial treatment was started in 10 cases. 6 patients were treated exclusively with ceftriaxone and 1 with both ceftriaxone and ciprofloxacin. 2 were initially treated with ciprofloxacin and 1 with piv-mecillinam. Once microbiological diagnosis was confirmed all but one were switched to ceftriaxone treatment. The median time of treatment was 14 days (range 10–28 days) for all cases. The median time of treatment with ceftriaxone was 10 days (range 3–14 days). Overall, 11 patients received oral treatment in the last part of the treatment course. Ciprofloxacin was administered in five cases; in one of these the bacteria was fully susceptible to ciprofloxacin and in the rest the isolates had reduced susceptibility. The remaining cases were treated with piv-mecillinam, pondocillin or sulphamethoxazol-trimethoprim.

Relapse was confirmed in 7 (37%) cases with a median of 20.5 days (range 13–27 days) between the last day of treatment and the day patients returned with a relapse. Patients were hospitalized with relapse for a median of 12 days (range 3–17 days). All patients with relapse were initially infected by a strain not fully susceptible to ciprofloxacin. All had been treated with ceftriaxone for a median of 10 days (range 4–14 days), 3 exclusively with ceftriaxone (10–14 days), 2 with ceftriaxone and ciprofloxacin, 1 with ceftriaxone and pondocillin and 1 with ceftriaxone, ciprofloxacin and piv-mecillinam.

Six cases were retreated with ceftriaxone between 10 and 14 days. Of these, 5 received additional treatment with amoxicillin, sulphametoxazol-trimethoprim or piv-mecillinam. One patient was retreated with a combination of piv-mecillinam, ciprofloxacin, and gentamicin. In total, the relapse was treated for a median of 40 days (range 10–42 days). One patient was diagnosed with gallstones and had an elective cholecystectomia. There was no second relapse and no fatal outcome.

12 cases were successfully treated and did not present with relapse. All but one received ceftriaxone and were treated with this for a median of 7 days (range 3–14 days) this being shorter than the cases in which relapse occurred. 8 out of 12 patients were infected with a strain with reduced sensitivity to ciprofloxacin.

## Discussion

Patients presenting with typhoid fever in high-income countries are usually young adults of 40 years of age or younger, which could simply reflect the age of travellers to endemic areas [3,10]. The VFR-group seems to be more vulnerable to typhoid fever than common tourists, as 89% of our population were VFR. None of the cases were infected in Denmark. Consistent with results of previous studies 84% of the cases in this study were infected while visiting the Indian subcontinent [9,10]. Between 1986–90 51% of the Danish cases were infected in this region [2]. Travelling to these destinations appears to be associated with increased risk of infection [2,4,12,14]. While the general risk of developing typhoid fever after travelling to a low-income country is estimated to be 1:100.000, the risk is as high as 30:100.000 on the Indian subcontinent [9]. In one British study, including both typhoid and paratyphoid fever, it was found that 84% were likely to have been infected in India and only 5.9% in Pakistan [11]. The majority were, as in this study and other studies, VFR-travellers [2,9,12,14]. The large number of patients being infected in India compared to present study could be explained by the relative larger population of people of Indian origin than of Pakistan origin in Great Britain compared to Denmark [15,16].

Differences in travel patterns might explain the increased number of infected among the VFR-travellers: It seems reasonable to assume that this group has less control over their diet and spend more time in rural areas living in close contact with the locals than other travellers. This would give them a risk profile similar to the locals [11]. It also makes them at greater risk of contraction other travel-related illnesses such as hepatitis A [17]. Therefore, family doctors should pay great attention to pre-travel advise directed at VFR-travellers regarding hygiene precautions and vaccine possibilities.

Even though vaccines on the market today only seem to prevent 70% of the cases [1], vaccination could be relevant for this specific group. In Australia, two studies of febrile travellers returning from abroad showed that typhoid fever was 10–11 times more likely in non-vaccinated individuals compared to those who received vaccination prior to travelling [18,19].

The patients in this study, in line with previous studies, sought medical attention within a month after returning from travelling [3,10].

68% of the patients were febrile at time of admittance; the rest reported to have experienced fever at some point in their illness. The reason why fever could not be documented in all cases could be explained by inaccuracy in the measurement as well as temperature fluctuation. The patients could also have been taking paracetamol prior to seeking medical attention without this being brought to the clinician’s attention. Despite this, the finding highlights that fever is the most common finding in patients affected with typhoid fever [12].

The clinical findings in this study were overall comparable to former studies where fever, headache and diarrhoea occurred with an incidence of 100%, 62-68% and 50-59%, respectively [3,10]. Typhoid fever should therefore be considered in all patients with a fever and a relevant travel history.

Even though gastroenteritis is not a key symptom, 84% of our cases presented with a gastrointestinal symptom in one form or another and such a finding should support the diagnosis.

Clinical findings traditionally associated with typhoid fever include rose spots, splenomegaly and relative bradycardia [4]. None of the patients in this study presented with rose spots and there were no reports on splenomegaly or relative bradycardia.

Splenomegaly and rose spots would most likely be found at the clinical examination when the patient was admitted. Relative bradycardia could be a symptom missed by the clinician.

In three studies including larger populations (100, 92 and 42 cases respectively) only 4.5-15% presented with splenomegaly, 4-9% with rose spots [3,10,12] and 13-27% with relative bradycardia. This indicates that these symptoms are not common in travellers and should only play a minor role in the diagnostics in high-income countries.

The most significant finding in the blood samples was the elevated LDH and CRP levels in all cases. This however cannot be used alone since LDH and CRP has been found elevated in blood samples from all febrile travellers in a previous study [13], as well as in malaria cases [20]. Because of this, these two parameters are not that useful to the clinician, since malaria is the most important differential diagnosis. In other studies ALAT has been found to be elevated with median levels from 56–75 U/L [10,13] or raised above normal in 82% of the cases [12]. In this study ALAT was elevated in 47% of the cases with a median level of 50 U/L when including both males and females.

Elevated ALAT at time of admittance should support the diagnosis. Serum sodium in the low end of normal range was found in comparable studies [3,10,12] as well as in this study.

All patients but one in this study received empirical treatment with ceftriaxone though it was not consistent for how long the treatment was maintained. Multi drug resistant (MDR) and ciprofloxacin resistant strains of *S.* Typhi is a common problem especially for travellers returning from the Indian subcontinent. Therefore ciprofloxacin should not be the drug of choice for empirical treatment [4]. It may only be considered for empirical treatment if the patient is contaminated *outside* the Indian subcontinent where the amount of resistant strains is still not critically high.

An alternative drug is azithromycin. It can be administrated orally and is therefore more convenient for the patient, especially in the last part of the treatment when hospitalization is no longer needed. Drug susceptibility test for azithromycin was not routinely performed on patient samples and might be worth considering in the future, since a cochrane-review from 2008 indicates that azithromycin could cause fewer cases with relapse than ceftriaxone [21]. This study however still needs to be performed on travellers [4].

In one review it was estimated that 5-10% of patients with enteric fever experienced relapse [4]. In a French study from 2001, relapse rate was 3% [3]. In the present study the relapse rate was 37%, which is an unexpected high rate.

It was not possible to determine a connection between the duration of treatment and the risk of relapse, and so, optimal length of treatment needs further investigation.

However, it is noticeable that none of the patients with relapse were infected by ciprofloxacin susceptible strains. In the French study only two patients were infected with strains resistant to ciprofloxacin [3]. This indicates that ciprofloxacin-resistant strains are more difficult to clear even with antibiotics that they are found to be sensitive for. Similar results have been found in another study [9], suggesting a connection between the high level of relapse and the rising resistance to ciprofloxacin. No connection was found between the risk of relapse and the onset of treatment with ceftriaxone [10].

Limitations to the present study include the low number of cases, as well as the fact that this study was done retrospectively and that the material does not allow for statistical testing of results. This however would not influence the data on relapse and para clinical findings.

Despite of limitations, this study provides a guide to which patients and symptoms might indicate typhoid fever. It is also consistent with previous national and international findings.

## Conclusions

Typhoid fever should be considered in young febrile adults returning from a VFR-trip to the Indian subcontinent, in a Danish setting, mainly Pakistan. Headache, gastrointestinal symptoms and elevated levels of LDH and CRP support the diagnosis.

Relapse was more frequent than expected and is associated with ciprofloxacin resistance. The doctor should be aware of this and take this into consideration when informing the patients. It is possible that azitromycin could reduce the rate of relapse, and this should be investigated further.

Finally, this study highlights the need for better pre-travel counselling of VFR-travellers.

## Competing interests

The authors have no conflicts of interest to declare and no involvement in any company with direct or indirect financial interest in the subject of the manuscript. No funding was received.

## Authors’ contributions

FCB collected data, analysed them and drafted the manuscript. JDK initiated the idea of looking into this specific group of patients, collected microbiological data and wrote parts of the manuscript concerning laboratory methods and revised the manuscript as a whole. ISJ drafted the design of the study, wrote parts of the manuscript, and revised it. All authors read and approved the final manuscript.
